# Editorial: Hormones and Neural Aging: Lessons From Experimental Models

**DOI:** 10.3389/fnagi.2018.00374

**Published:** 2018-11-15

**Authors:** Isabel Varela-Nieto, Julie A. Chowen, Luis Miguel García-Segura

**Affiliations:** ^1^“Alberto Sols” Biomedical Research Institute, CSIC-UAM, Madrid, Spain; ^2^Centro de Investigación Biomédica en Red de Enfermedades Raras, Instituto de Salud Carlos III, Madrid, Spain; ^3^Hospital La Paz Institute for Health Research (IdiPAZ), Madrid, Spain; ^4^Department of Endocrinology, Hospital Infantil Universitario Niño Jesús, Instituto de Investigación la Princesa, Madrid, Spain; ^5^Centro de Investigación Biomédica en Red de Fisiopatología de la Obesidad y Nutrición (CIBEROBN), Instituto de Salud Carlos III, Madrid, Spain; ^6^IMDEA Food Institute, CEI UAM + CSIC, Madrid, Spain; ^7^Instituto Cajal, CSIC and Centro de Investigación Biomédica en Red de Fragilidad y Envejecimiento Saludable, Instituto Carlos III, Madrid, Spain

**Keywords:** apoptosis, insulin-like factors, neurodegeneration, senescence, sex hormones

The relationship between the hormonal status and neural aging has been studied in different contexts, from metabolism to reproduction. Age-related modifications in circulating hormones that perform neuroprotective functions have been associated with neural aging. In addition, the sensitivity of neurons and glial cells to respond to some peripheral metabolic signals and neuroprotective substances declines with aging. In turn, neural aging affects the control exerted by the hypothalamus on peripheral endocrine glands and systemic metabolism. Therefore, the feed-back loops between the brain and the body are progressively altered during the aging process, contributing to neural dysfunction.

The study of aging by using experimental models offers an opportunity to better understand human physiopathology, with these studies complementing clinical and epidemiological studies. This collection of articles attempts to portray how animal studies have advanced our current knowledge on the interaction of hormones with neurodegeneration changes, including those in sensory systems, that take place during aging. These articles discuss the molecular pathways implicated in the decreased cell renewal, cell senescence and cell death, as well as potential strategies to promote healthy brain aging and rejuvenation.

In the review by Arroba et al., age-related neurodegenerative diseases of the retina are discussed. Neuroinflammation is one of the hallmarks of these diseases, including age-related macular degeneration, retinitis pigmentosa, and diabetic retinopathy. Insulin-like growth factor (IGF)-1 plays numerous roles in the retina and the involvement of this growth factor, as well as microglia and exosomes, in these neurodegenerative processes is highlighted. Mukai et al. present original data regarding how the complement system is involved in maintaining retinal integrity during aging. Various complement knock-out strains of mice were employed. Electroretinograms (ERG) and thicknesses of retinal layers in spectral domain optical coherence tomography were determined in young and adult wild type and C*1q*^−/−^, *Mbl a/c*^−/−^, *Fb*^−/−^, *C3*^−/−^, and *C5*^−/−^ knock-out mice. The data presented support the hypothesis that the complement system is important in maintaining retinal integrity during aging.

Hearing function is also affected during aging and Balogová et al. present an original study employing the fast aging Fischer 344 rat model. They compared age-related changes in cochlear morphology, hearing thresholds, auditory brainstem responses, and distortion product otoacoustic emissions in both male and female rats, and found that the age-related changes in hearing function are more pronounced in males compared to females. A mini review by Rodríguez-de la Rosa et al. delves into the role of IGF-1 in age-related hearing loss and the mechanisms involved. This group has reported a correlation between the decline in IGF-1 availability and the reduction in hearing during aging. The primary actions of IGF-1 on audition and the mechanisms involved are discussed.

The mechanisms of neuroprotective effects of IGF-1 are reviewed in depth by Labandeira-Garcia et al. The role of this growth factor in protection against neuroinflammation and the possible explanations for some of the controversies surrounding this topic are presented. The implication of astrocytes and microglia in IGF-1 mediated neuroprotection, such as their production of local IGF-1 and the cross-talk between these glial cells and neurons, is discussed. Importantly, these authors also review the interaction between IGF-1 and estrogens in neuroprotection.

Estrogens exert important neurotrophic and neuroprotective effects and the decline in this sex steroid is suggested to be involved in some of the aging processes in women. Various articles in this collection focus on the role and mechanisms of estrogens in the aging brain. In the review by Lejri et al., the relationship between estrogens and mitochondrial function in the aging brain is discussed. Indeed, mitochondria are essential for the production of sex steroids and, in turn, these hormones can modify mitochondrial function. Here, mitochondrial dysfunction during aging is described, as well as how estrogens affect this process. Special emphasis on recent evidences in the involvement of brain-derived neurotrophic factor and sirtuin 3 (SIRT3) in the neuroprotective effects of estrogens, especially in female aging, is made. This topic is also reviewed by Gaignard et al., where they analyze in depth the intracellular mechanisms of sex steroid actions on mitochondrial function. These authors also address aspects of sexual dimorphism in mitochondria during processes of aging and how this might be involved in the sex differences in age-related neurodegenerative diseases. The mechanisms by which sex steroids promote neuroprotection have been analyzed in the review by Zárate et al. These authors discuss the effects of sex steroids on DNA repair enzymes and how a reduction in estrogens in postmenopausal women may be involved in the reduced capacity for DNA repair that occurs in aging processes.

Women's health during aging is also addressed in the review by Navarro-Pardo et al. Here, the authors concentrate on the important question of how declining circulating estrogen levels during aging affect cognitive function and psychological health in women. The debate as to whether hormone therapy is beneficial in postmenopausal women is also examined. A study analyzing the connection between adrenomedullin levels and memory preservation during aging by Larrayoz et al. show an inverse relationship between these two factors. Knock-out mice for adrenomedullin and its generated peptide, promadreullin N-terminal 20 peptide, were employed and memory retention was found to be improved in these knock-out mice compared to wild type controls, with this effect being more prominent in females compared to males. This improvement was associated with decreased phospho-Tau accumulation. They also report that adrenomedullin accumulation increases with aging in human brain suggesting that this hormone may be a target for improving age-related memory decline.

A relationship between cognitive decline during aging and the hypothalamic-pituitary-adrenal (HPA) axis has been hypothesized. Pesce et al. present an original article where they assessed the effects of memory training on cognitive performance and the HPA axis, as well as systemic inflammation, in human subjects. Young and older adults were analyzed at baseline and the young adults were also analyzed 6-months after memory training. These authors report an improvement in cognitive function, memory, and attention by memory training that was associated with a decrease in circulating levels of cortisol and inflammatory cytokines. They also raise the question as to whether the wild type p53-induced phosphatase-1 (WIP-1) is involved in this process, as its expression in mononuclear cells was positively associated to the cognitive function scores.

Together these articles indicate that, although numerous factors are involved in the aging process, the decline in endocrine function appears to play a very important role in neural aging. Aging of the central nervous system clearly impacts the physiological control of the entire being, including a decline or alteration in endocrine system function, which in turn would contribute to further neural aging. Further studies employing animal models are necessary to understand how to slow this vicious cycle.

## Author contributions

IV-N, JAC, and LG-S contributed equally to this editorial, in its conception, writing, and revision.

### Conflict of interest statement

The authors declare that the research was conducted in the absence of any commercial or financial relationships that could be construed as a potential conflict of interest.

